# Protein Corona of Anionic Fluid-Phase Liposomes Compromises Their Integrity Rather than Uptake by Cells

**DOI:** 10.3390/membranes13070681

**Published:** 2023-07-20

**Authors:** Daria Tretiakova, Maria Kobanenko, Anna Alekseeva, Ivan Boldyrev, Sergey Khaidukov, Viktor Zgoda, Olga Tikhonova, Elena Vodovozova, Natalia Onishchenko

**Affiliations:** 1 Laboratory of Lipid Chemistry, Department of Chemical Biology of Glycans and Lipids, Shemyakin–Ovchinnikov Institute of Bioorganic Chemistry, Russian Academy of Sciences, ul. Miklukho-Maklaya 16/10, 117997 Moscow, Russia; daria@lipids.ibch.ru (D.T.); mkobanenko@lipids.ibch.ru (M.K.); anna@lipids.ibch.ru (A.A.); ivan@lipids.ibch.ru (I.B.); elvod.ibch@yandex.ru (E.V.); 2 Laboratory of Carbohydrates, Department of Chemical Biology of Glycans and Lipids, Shemyakin–Ovchinnikov Institute of Bioorganic Chemistry, Russian Academy of Sciences, ul. Miklukho-Maklaya 16/10, 117997 Moscow, Russia; 3 Institute of Biomedical Chemistry, ul. Pogodinskaya 10, 119121 Moscow, Russia; vic@ibmc.msk.ru (V.Z.); ovt.facility@gmail.com (O.T.)

**Keywords:** liposomes, fluid-phase bilayer, protein corona, cell uptake

## Abstract

Despite the undisputable role of the protein corona in the biointeractions of liposome drug carriers, the field suffers from a lack of knowledge regarding the patterns of protein deposition on lipid surfaces with different compositions. Here, we investigated the protein coronas formed on liposomes of basic compositions containing combinations of egg phosphatidylcholine (PC), palmitoyloleoyl phosphatidylglycerol (POPG), and cholesterol. Liposome−protein complexes isolated by size-exclusion chromatography were delipidated and analyzed using label-free LC-MS/MS. The addition of the anionic lipid and cholesterol both affected the relative protein abundances (and not the total bound proteins) in the coronas. Highly anionic liposomes, namely those containing 40% POPG, carried corona enriched with cationic proteins (apolipoprotein C1, beta-2-glycoprotein 1, and cathelicidins) and were the least stable in the calcein release assay. Cholesterol improved the liposome stability in the plasma. However, the differences in the corona compositions had little effect on the liposome uptake by endothelial (EA.hy926) and phagocytic cells in the culture (U937) or ex vivo (blood-derived monocytes and neutrophils). The findings emphasize that the effect of protein corona on the performance of the liposomes as drug carriers occurs through compromising particle stability rather than interfering with cellular uptake.

## 1. Introduction

The concept of biomolecular corona as a natural interface adopted by nanoparticles in biological media has been dominating the field of nanoparticulate drug delivery systems since it was introduced [1,2]. The layer of proteins, as well as other biomolecules deposited on the surface of the nanoparticles in the plasma and other bodily fluids, is determined by the nanoparticle composition and has been shown to be involved in nanoparticle–cell interactions, affecting the biodistribution of the carrier, internalization rates by cells, and intracellular trafficking [3,4]. As pioneers of clinically approved drug delivery vehicles, liposomes still represent attractive carriers due to their biocompatibility [5], robust technologies of production [6], and versatility. Precise knowledge of the laws regarding the formation of protein corona and its effects on clinical outcomes could provide guidance for the entire field of nanomedicine. Nevertheless, studies on the protein corona of lipid-based nanoparticles are considerably underrepresented in nanobiointerface research [7].

In early works, the liposome circulation time was shown to be dependent on the lipid composition, which determines the fluidity and surface charge of the bilayer [8]. Fluid-phase liposomes have long been known to be rapidly cleared from circulation, which correlated with rather high amounts of bound plasma proteins [8] (this has kept the interest in fluid phase-based liposomes as drug vehicles relatively low). The integrity of liposomes in the presence of plasma proteins due to the robustness of the solid-phase (and even more so liquid ordered-phase) bilayer does have a flip side, namely slower intracellular processing [9]; neither was it found to be beneficial for hydrophobic drugs incorporated in the lipid bilayer of liposomes, which escape solid phase-based liposomes in the plasma more easily [10]. While some negatively charged lipids increase protein binding (PB) by liposomes and, thus, decrease the clearance time, phosphatidylglycerol affects the clearance but not the PB value [11]. Cholesterol, through modulating the phospholipid packing, membrane rigidity, and fluidity, was shown to increase the liposome stability, which often positively affects drug retention [12,13]. It was further shown to increase the encapsulation efficiency of doxorubicin for lipid compositions comprising of saturated phospholipids; however, it failed to do so in a mixture with egg phosphatidylcholine (PC) and ~5% of DSPE-PEG2000 in the bilayer [14]. The inclusion of a PEG2000–saturated phospholipid conjugate in a cholesterol-containing tightly packed lipid bilayer is a common method to stabilize liposomes in the bloodstream through shielding the membrane with highly hydrophilic polymer chains [15,16]. Multiple interactions of ethylene glycol residues of PEG with water molecules result in a highly structured solvent shell surrounding the bilayer that is difficult to displace for the proteins. However, anti-PEG antibodies are the major reason for the accelerated blood clearance effect of PEG-bearing formulations and can cause serious side effects [17,18]. Utilization of fluid-phase bilayers, including ones with anionic lipids, to address the current challenges in liposomal drug delivery requires a better understanding of their interactions with plasma proteins, which has remained understudied so far.

In the past 15 years, the evolution of shotgun proteomics methods has added a new dimension to the protein corona [19,20,21,22,23,24,25,26]. The improved resolution resulted in the expansion of the liposome-associated protein list to hundreds of titles. This, in turn, led to the first use of bioinformatics to predict liposomal behavior based on the composition of the protein corona [27]. However, only fragments of the vast space of lipidic compositions have been explored thus far, mainly focusing on clinically approved formulations, such as DOXIL and Ambisome, and often lipoplexes. This was accompanied by an inconsistent approach to the isolation of liposome-associated proteins from their mixtures with plasma, often leading to considerable and uncontrolled contamination with other biological entities of a similar size and density as the liposomes and, thus, to contradictory results [28].

We initiated this study on plasma protein binding by basic lipid compositions as part of a larger effort to elucidate the trends in how liposome composition determines plasma protein adsorption in a well-controlled and consistent experimental setup. In this paper, we investigated the impact of a negatively charged phospholipid, phosphatidylglycerol (PG), and/or cholesterol on plasma protein absorption by fluid-phase liposomes. We started with isolation of the liposome−protein complexes and analysis of the protein corona composition with LC-MS/MS. We then compared liposome stability in the presence of plasma proteins with respect to cargo release. Finally, we assessed the liposome uptake by cultured and isolated blood cells as the basic functional effects of protein binding.

## 2. Materials and Methods

### 2.1. Chemicals and Materials

Egg phosphatidylcholine (ePC; USP grade, Lipoid E PC S) and sheep wool cholesterol (Chol, USP grade) were obtained from Lipoid GmbH (Heidelberg, Germany); 1-palmitoyl-2-oleoyl-sn-glycero-3-phospho-(1’-rac-glycerol) (POPG, sodium salt) was obtained from Avanti Polar Lipids Ltd. (Birmingham, AL, USA). 1,3,5,7-Tetramethyl-BODIPY-labeled phosphatidylcholine (TMB-PC) was synthesized as previously reported [29]. All buffer components were purchased either from Helicon (Moscow, Russia) or PanEco (Moscow, Russia), Sepharose CL-4B for size-exclusion chromatography (SEC), from Sigma (Burlington, MA, USA). Chloroform and other solvents were purified according to standard procedures.

Buffer compositions were as follows: phosphate-buffered saline (PBS; KH2PO4, 0.2 g/L; NaH_2_PO4 × 2H_2_O, 0.15 g/L; Na_2_HPO_4_, 1.0 g/L; KCl, 0.2 g/L; NaCl, 8.0 g/L, pH 7.4); Tris-buffered saline (TBS; NaCl, 4.39 g; Tris, 3.03 g; H_2_Odd, 500 mL), pH 7.97; Tris-HCl, pH 7.0 (30 mM Tris); SDS-PAGE sample buffer (0.075 M Tris-HCl, pH 6.8, 10% glycerin, 2% SDS, 5% β-mercaptoethanol, 0.01% bromophenol blue).

Primary polyclonal rabbit antibodies to human serum albumin HSA (IMTEK, Moscow, Russia) and to human ApoH (Cloud-Clone Corp., Houston, TX, USA); monoclonal murine antibodies to immunoglobulins M and to ApoA1 (IMTEK, Moscow, Russia) were used. Secondary antibodies were rabbit antibodies to mouse IgG and mouse antibodies to rabbit IgG conjugated with horseradish peroxidase (Sigma-Aldrich, St. Louis, MO, USA).

Blood samples from nine healthy donor volunteers were collected in vacuum tubes over EDTA (Greiner Bio-One, Kremsmünster, Austria). Plasma was separated by centrifugation at room temperature for 30 min at 1660× *g* (CM-6M, ELMI, Riga, Latvia). The supernatants were pooled, transferred into fresh tubes, and centrifuged at 600× *g* for another 10 min (CM-6M, ELMI, Riga, Latvia). Plasma aliquots were frozen in liquid nitrogen and stored at –70 °C. For the experiments, an aliquot of plasma was thawed at 37 °C and used immediately.

### 2.2. Liposome Preparation

Liposomes (large unilamellar vesicles) were prepared by lipid film hydration followed by extrusion. Mixtures of ePC, Chol, and POPG in the required molar ratios (see Table 1 for liposome compositions) were co-evaporated from solutions in chloroform in round-bottom tubes on a rotary evaporator. The lipid films were further dried on an Iney-4 (Institute for Biological Instrumentation, Russian Academy of Sciences, Pushchino, Russia) freeze dryer at 7 Pa and hydrated with PBS (unless otherwise indicated) at room temperature for 2 h with stirring. Then, the mixtures were subjected to 5–7 cycles of freezing (N_2_ liquid)–thawing (+40 °C) and extruded through Whatman Nuclepore membrane filters (Cytiva, Marlborough, MA, USA) with calibrated pore size of 100 nm 20 times using an Avanti Polar Lipids (USA) mini-extruder. The resulting dispersions were stored at +4 °C and were used for experiments within 3 days.

To obtain fluorescently labeled liposomes for cell internalization experiments, 1 mol% TMB-PC was added at the stage of lipid film formation.

### 2.3. Liposome Characterization

Phospholipid concentrations in liposome dispersions were measured by the enzymatic colorimetric phosphatidylcholine assay (Sentinel Diagnostics, Milan, Italy). Particularly, 3 μL of a fraction and 150 μL of the working enzyme solution (phospholipase D, >1500 U/L; choline oxidase, >7500 U/L; 4-aminoantipyrine, 1.2 mM; peroxidase, >7000 U/L; TES, 50 mM, pH 7.6; hydroxybenzoic acid 12 mM; EDTA, 1.3 mM; sodium azide, <0.1%) were added per well in a 96-well plate. The mixture was incubated at 37 °C for 10 min. Optical density was read at 540 nm using a Multiskan FC (Thermo Fisher Scientific, Waltham, MA, USA) microplate photometer. The amount of phosphatidylcholine in the samples was determined using the calibration curve for PC dispersions in PBS.

To determine hydrodynamic diameter of the liposomes, the dispersions were diluted to a final lipid concentration of 50 µg/mL in PBS. The measurements were carried out on a Brookhaven Particle Analyzer 90+ (Brookhaven Instruments Corp., Holtsville, NY, USA; helium-neon laser, 633 nm, 90° angle), 3 cycles of 1 min, or Zetasizer Nano ZS (Malvern Panalytical, Ltd., Malvern, UK; 633 nm, 173° angle; provided by the BioImaging and Spectroscopy Core Facility of the Skolkovo Institute of Science and Technology, Moscow, Russia), 3 measurements of 10 cycles per sample.

For reliable measurements of zeta potential, liposome samples with diameters of around 200 nm were prepared in 10 mM KCl, 1 mM K_2_HPO_4_, 1 mM KH_2_PO_4_, pH 7.0 buffer (extruded 20 times through 200 nm polycarbonate membrane filters). Samples of the liposomes (0.85 mL, 1 mg/mL total lipids) were equilibrated for 1 min in cuvettes before a measurement of 100 to 500 cycles per sample were performed at 25 °C using Litesizer 500 (Anton Paar GmbH, Graz, Austria; 658 nm). Measurements were taken in triplicate.

### 2.4. Isolation of Liposome–Protein Complexes for Zeta Potential Determination

Liposomes prepared in 10 mM KCl, 1 mM K_2_HPO_4_, 1 mM KH_2_PO_4_, pH 7.0 buffer, approximately 200 nm in size were used. A 200 μL aliquot of frozen pooled plasma was thawed on a water bath at 37 °C for 15 min and centrifuged at 12,000× *g* for 30 min. Then, 90 μL of supernatant was mixed with 90 μL liposome dispersion (20 mM) and incubated at 37 °C for 15 min. After the incubation, proteolysis was stopped by adding ethanol solution of phenylmethylsulfonyl fluoride (0.1 M, 1.8 μL) and 180 μL of thus obtained dispersion was applied onto a Sepharose CL-4B column (1.0 × 27 cm) equilibrated with 10 mM KCl, 1 mM K_2_HPO_4_, 1 mM KH_2_PO_4_, pH 7.0 buffer. After elution of the void volume (~7 mL), 16 fractions of ~200 mL were collected. Liposome and protein elution was monitored by absorbance at 210 and 280 nm using the NanoDrop OneC spectrophotometer (Thermo Fisher Scientific, Waltham, MA, USA). Five fractions most rich with liposomes were pooled, mixed, and transferred to disposable Omega cuvette (Anton Paar GmbH, Graz, Austria) without dilution to obtain zeta-potential value. For each liposome sample, incubation and further treatment were performed thrice.

### 2.5. Liposome Stability Assay

The stability of liposomes in the presence of human blood plasma was investigated using the dye leakage method as previously described [30]. To prepare liposomes with calcein in a self-quenching concentration, lipid films were hydrated with 80 mM calcein solution in PBS. After extrusion, unencapsulated calcein was separated from calcein-containing liposomes using size exclusion chromatography on a column with Sephadex G-50 (1.3 × 18 cm) equilibrated in PBS. An aliquot of liposome dispersions (200 μL) was applied to the column and after elution of most of the void volume (~4.5 mL), fractions of 150–200 µL were collected. Fractions with the highest liposome content were pooled. Calcein concentration in combined fractions was determined by spectrophotometry (λ_max_ 504 nm, ε 74,000 M^–1^ cm^–1^).

An aliquot of calcein-containing liposomes was diluted with pre-heated (37 °C) PBS or 50% human blood plasma (pre-treated as described in Section 2.4) to a concentration of 10^–4^–10^–5^ M and incubated at 37 °C for 0, 0.5, 1, 2, or 4 h. The fluorescence intensity of calcein was determined using the temperature-controlled cell of an F-4000 (Hitachi, Japan) fluorescence spectrometer before and after the liposome lysis with 30 μL of 10% Triton X-100 solution added per 300 μL of dispersion, λ_ex_ 485 nm, λ_em_ 509 nm. The fraction of calcein released before the addition of the detergent was calculated using the formula:

CR = (*I*_t_/*I*_max,t_ − *I*_0_/*I*_max,0_) × *I*_max,t_/(*I*_max,t_ − *I*_0_) × 100% (1)

where CR is calcein release; *I*_max,0_ is the fluorescence intensity upon Triton X-100 addition immediately after the liposome dilution; *I*_max,t_, fluorescence intensity upon Triton X-100 addition to diluted liposomes after incubation for time t. The CR values obtained in triplicate were used to plot the relative increase in the fluorescence of calcein in function of the incubation time.

### 2.6. Isolation of Liposome–Protein Complexes for LC-MS/MS

A 600 μL aliquot of frozen pooled plasma was thawed on a water bath at 37 °C for 15 min and centrifuged at 12,000× *g* for 30 min. Then, 540 μL of supernatant was mixed with 60 μL liposome dispersion (40 mM) and incubated at 37 °C for 15 min. After the incubation, proteolysis was stopped by adding ethanol solution of phenylmethylsulfonyl fluoride (0.1 M, 6 μL) and 500 μL of thus obtained dispersion was applied onto a Sepharose CL-4B column (1.5 × 33 cm) equilibrated with PBS. After elution of most of the void volume (~15 mL), 16 fractions of ~1 mL were collected. Liposome and protein elution was monitored as described in Section 2.4. Four fractions most rich with liposomes were pooled and concentrated (in several aliquots) down to 120 μL using the Vivaspin 2 concentrators (MWCO 300 kDa, Sartorius, Germany) by centrifugation for ~100 min at 740× *g* (CM-6M, ELMI) at room temperature. Before usage, concentrators` membranes were passivated with 1% BSA solution overnight at 4 °C and washed as specified by the manufacturer.

As a negative control (when no protein corona is formed), a plasma sample was treated in the same manner as the liposome–protein mixtures. Upon SEC separation of the plasma control, protein concentration in fractions corresponding to the liposome elution was too low for a proteomics analysis (see further below). So, we combined the material of three SEC elutions of control plasma.

### 2.7. HPLC-MS/MS Sample Preparation

Samples of liposome–protein complexes were hydrolyzed with trypsin according to the S-Trap (suspension trapping proteolysis) protocol [31]. To the samples, 20 μL 100 mM triethylammonium bicarbonate (TEAB) buffer was added and mixed with lysing buffer (10% SDS, 100 mM TEAB, pH 7.55) 1:1. The dispersion was sonicated for 20 s thrice and centrifuged at 13,000× *g* for 8 min at 10 °C. Then, 2 μL 0.5 mM tris(2-carboxyethyl)phosphine hydrochloride (Sigma-Aldrich, St. Louis, MO, USA) and 4 μL 400 mM 2-chloroacetamide were added. The samples were incubated for 30 min at 80 °C and cooled to room temperature. After the addition of 12% H_3_PO_4_ at 1:10 ratio, the dispersion was pipetted and 6-fold volumes of S-Trap protein binding buffer (90% aqueous methanol containing 10 mM TEAB, pH 7.5) were added. The resulting mixture was shaken thoroughly and applied onto an S-trap filter and centrifuged for 4 min at 4000× *g*. The sample was washed with 150 μL S-Trap protein binding buffer 4 times at 4000× *g*. Trypsin solution in 50 mM TEAB was added to the sample (trypsin to protein ratio of 1:25) and incubated for 1.5 h at 47 °C. After the incubation, 40 μL 50 TEAB and 0.2% formic acid were added and the sample was centrifuged for 4 min at 4000× *g*. Then, 35 μL 0.2% formic acid solution in 50% acetonitrile was added and centrifuged for 4 min at 4000× *g*. The hydrolyzates (supernatants) were dried in a vacuum concentrator (Eppendorf Concentrator plus, Hamburg, Germany) and redissolved in 0.1% formic acid to a final concentration of 1 μg/μL. peptide concentration in samples was assayed using the Pierce™ Quantitative Colorimetric Peptide Assay kit (Thermo Scientific, Waltham, MA, USA) according to the manufacturer’s recommendations.

### 2.8. HPLC-MS/MS Data Acquisition and Analysis

Proteomic analysis of peptide mixtures was performed using the Ultimate 3000 RSLCnano (Thermo Scientific, Waltham, MA, USA) system equipped with a Q-Exactive HFX (Thermo Scientific) mass spectrometer. One microliter of the peptide mixture was applied to an Acclaim µ-Precolumn (0.5 × 3 mm, 5 μm) at a flow rate of 10 μL/min for 4 min under isocratic mode using buffer C as a mobile phase (2% acetonitrile, 0.1% formic acid in deionized water). Then, the peptides were separated using the Acclaim Pepmap^®^ C18 (75 μm × 150 mm, 2 μm) (Thermo Scientific) column under the following gradient of B (80% acetonitrile in 0.1% aqueous formic acid) in A (0.1% aqueous formic acid): 2% for 10 min, from 2 to 35% for 68 min; from 35 to 99% for 2 min; 99% for 2 min; from 99 to 2% for 3 min. Total duration of the analysis was 90 min.

Mass spectrometry analysis was performed under positive ionization mode using the NESI source (Thermo Scientific). The following settings were used: emitter voltage 2.1 kV; capillary temperature 240 °C. Panoramic scanning was performed in the mass range from 300 to 1500 *m*/*z* at 120,000 resolution. For tandem scanning, 15,000 resolution in the mass range from 100 to the upper limit determined automatically from precursor mass, but not more than 2000 *m*/*z* was used. Precursor ions were isolated in the ±1 Da window. The maximum number of resolved ions under the MS2 mode was set as not more than 40, the limit of precursor choice for tandem analysis was set at 50,000 units and normalized collision energy (NCE) was 29. For tandem scanning, only ions with *z* form 2+ to 6+ were considered. The maximum time for precursor ion accumulation was 50 ms, and for fragment ions, 110 ms. The AGC (Automatic Gain Control) value for precursor and fragment ions was 1 × 10^6^ and 2 × 10^5^, respectively. All measured precursors were dynamically excluded from tandem MS/MS analysis for 90 s.

Raw MS data files were analyzed using the MaxQuant v. 2.1.0.0 software with the Andromeda searching algorithm [32]. The UniProt FASTA human protein database (April, 2022) was used for identification. The following search parameters were used: cleaving enzyme, trypsin; two missed cleavages allowed; monoisotopic peptide mass determination precision, ±4.5 ppm; mass determination precision in MS/MS spectra, ±20 ppm. Oxidation of methionines and acetylation at N terminus were considered possible and cysteine carbamidomethylation, necessary modification. For validation of peptide-spectrum matches, FDR (false discovery rate) <0.1% was used for peptide and protein identifications. Proteins were considered reliably identified if at least two peptides were detected. LFQ (label-free quantification) and iBAQ (intensity-based absolute quantification) values were used to evaluate protein quantities.

To evaluate content of the proteins in each experimental group of samples, the relative protein abundance values, RPA (%), were calculated based on the iBAQ values. For this, sum of the iBAQ values in the technical replicates for each protein was divided on the sum of iBAQ values for all identified proteins in the sample and multiplied by 100%. Statistical analysis of proteomic data was carried out using the Perseus v.1.6.15.0 software. To compare proteins between samples, the protein iBAQ (to calculate relative protein abundance, RPA, %) or LFQ (to compare protein intensities between samples) values of the samples were loaded into the program, including technical replicates. Six groups were formed: control plasma, PC, 10PG, 40PG, 33CH, and CHPG (3 replicates for 3 independent samples each). The data were filtered out beforehand: possible contaminant proteins and false positive identifications were removed; proteins identified by two or more peptides and present in at least 70% of samples in total were left for analysis. To determine statistically significant differences between the groups, the multi-sample ANOVA with post hoc Tuckey’s HSD test (FDR 0.05) was used. Statistically significant differences were considered with a fold change > 2 and a value of q < 0.01 with permutation-based FDR correction for multiple hypothesis testing.

### 2.9. Delipidization of Liposome–Protein Complexes, SDS-PAGE, and Immunoblotting

Delipidization was performed as described in [33]. To 100 μL of the combined fractions, 400 μL of cooled methanol was added and the mixture was centrifuged for 3 min at 9000× *g* (Eppendorf, Hamburg, Germany). To the solution, 200 μL of chloroform was added, vigorously stirred, and centrifuged for 3 min at 9000× *g*. To the mixture, 300 μL of water was added, vigorously stirred, and centrifuged for 4 min at 9000× *g*. Approximately 700 μL of the upper aqueous phase was discarded. Then, 300 μL of methanol was added to the residue and the mixture was centrifuged for 4 min at 9000× *g*. The supernatant was decanted, and the precipitate was evaporated to dryness on a rotary evaporator. The samples were dissolved in 36 μL of 2× reducing buffer (0.075 M Tris-HCl, pH 6.8, 10% glycerol, 2% SDS, 5% β-mercaptoethanol, 0.01% bromophenol blue), stirred, and boiled 2 × 2 min. SDS-PAGE was performed in 6% concentrating and 12% separating gels on a Mini Gel Tank (Thermo Fisher Scientific, Waltham, MA, USA) apparatus for 45 min at 200 V. Precision Plus ProteinTMDual Color Standards (Bio-Rad Laboratories, Inc., Hercules, CA, USA) was used as molecular weight marker. Proteins were visualized by silver staining or transferred to a PVDF membrane using the Mini Gel Tank (Thermo Fisher Scientific, Waltham, MA, USA) at 20 V for 60 min. After the end of the transfer, the membrane was washed with TBS and, to prevent non-specific sorption, incubated in 5% low-fat dry milk in TBS with 0.1% Tween 20 (TBS/T) for 1 h at room temperature. Then, the membrane was washed with TBS/T (3 × 5 min) and incubated with anti-IgM, HSA, ApoA1, ApoH primary antibodies in 0.5% BSA solution for 2 h at room temperature with stirring. The membrane was washed with TBS/T for 15 min and 3 × 5 min and incubated with horseradish peroxidase-conjugated secondary antibodies at 4 °C overnight. The membrane was then washed again with TBS/T 5 × 5 min. Immunodetection was performed using Clarity™ ECL Western Blotting Substrate (Bio-Rad) and VersaDoc 4000 (Bio-Rad).

### 2.10. Internalization by Cultured Cells

For the experiments, 10 mM total lipid liposomes with 1 mol. % BODIPY-labeled probe (TMB-PC) were used. A 600 μL aliquot of frozen pooled plasma was thawed on a water bath at 37 °C for 15 min and centrifuged at 12,000× *g* for 30 min. Then, 540 μL of supernatant was mixed with 60 μL liposome dispersion and incubated for 37 °C for 15 min. Immediately after that, the liposome–plasma mixtures were diluted in serum-free RPMI 1640 medium (PanEko, Moscow, Russia) to 100 μM total lipid concentration (plasma final concentration in the medium ~10%). Liposome−protein complexes were not separated from plasma before the addition to culture medium.

Human histiocytic lymphoma monocyte U937 cells were cultured in RPMI 140 medium supplemented with 10% fetal bovine serum (FBS), 2 mM L-glutamine, 100 U/mL penicillin, and 100 μg/mL streptomycin at 37 °C and 5% CO_2_. Cell suspensions 10^6^ cells/mL were washed with the medium (centrifugation for 5 min at 500× *g*), transferred into serum-free medium, and treated with liposomes of various compositions with and without the protein corona. Control samples contained liposome-free PBS or human plasma.

Human endothelial EA.hy926 cells were cultured in DMEM (PanEko) supplemented with 10% FBS, 2 mM L-glutamine, 100 U/mL penicillin, and 100 μg/mL streptomycin. Confluent cell monolayer in 24-well plates was washed with cold DPBS (PBS with calcium and magnesium salts, PanEco, Russia) and incubated with liposomes (with or without the protein corona) for various incubation periods. Then, the cells were washed with DPBS, resuspended in 0.02% EDTA solution (10 min, 37 °C).

Cell suspensions were stored on ice prior to measurements on a FACScan (Becton Dickinson, Franklin Lakes, NJ, USA) flow cytometer using the CellQuest software. Fluorescence signal was detected in channels FL1 (515–545 nm), FL2 (565–610 nm), and FL3 (>650 nm), two runs of 10,000 target events each per sample. To exclude cell aggregates, debris, or dead cells from the analysis, target events were gated by forward and side scattering (FSC/SSC) and the propidium iodide signal (0.3 μg/mL PBS solution of propidium iodide was added after final wash).

### 2.11. Internalization Ex Vivo by Blood Cells

Blood sample of healthy volunteer donor was collected in test tubes over lithium heparin as an anti-coagulant (Vacuette, Greiner Bio-One, Frickenhausen, Germany) and used on the same day. In total blood samples of 3, volunteers were used and the experiment was conducted thrice with the new donor blood for each repeat.

An aliquot of 5 μL of a 10 mM liposome sample with TMB-PC fluorescent probe was added to 100 μL of whole blood, mixed, and incubated at 37 °C for 15, 30, or 60 min. As a control, 5 μL of PBS was added to 100 μL of whole blood. After incubation, the samples were diluted with 3 mL of cold PBS (+4 °C) to stop phagocytosis, intensively stirred, and centrifuged for 10 min at 300× *g*. The supernatant was discarded, 1 mL of freshly made lysing buffer for erythrocytes (NH_4_Cl, 155 mM; NaHCO_3_, 12 mM; EDTA, 0.1 mM) was added, and the mixture was stirred and left for 1 h in the dark at room temperature (stirring was repeated every 20 min). Afterwards, samples were centrifuged for 10 min at 300× *g* and washed with 1 mL of PBS twice. Immediately before the measurements, to quench the fluorescence on the cell surface, an aliquot of crystal violet in PBS was added to its final concentration 11 μg/mL.

Stained cell measurements were performed on a Cytomics FC500 flow cytometer (Beckman Coulter, FL, USA) as in [34]. Fluorescent signal was detected in the FL1 channel using the 500–550 nm filter. The boundaries of positive and negative cells were defined on the fluorescence distribution histograms so that the main pool of negative cells remained in the first decade of the logarithmic scale in accordance with the control experiments (0 min incubation). The target peripheral blood leukocyte populations (lymphocytes, monocytes, and granulocytes) were detected by introducing logical constraints into cell distribution histograms for small-angle (forward scatter) and lateral (side scatter) light scattering using standard FACS analysis criteria [35]. Each population was individually analyzed using fluorescence of at least 10^5^ cells. The control experiment showed that identification of blood cell subpopulations by morphological parameters yielded the same results as staining with the CD45 leukocyte marker. The collected data were processed using the CXP analysis software package (Beckman Coulter, USA).

### 2.12. Statistical Analysis of Experimental Data

Unless otherwise indicated, experimental data were analyzed using QtiPlot 0.9.8.9. svn 2288 software by Ion Vasilief. Data obtained in Section 2.5, Section 2.6 and Section 2.11 were expressed as mean ± standard deviation (SD) to show dispersion of the experimental values. To evaluate whether internalization of liposome samples by cells depends on liposome composition, we used a two-sample *t*-test with the following parameters: independent test, significance level 0.05; confidence intervals at 95%. For Section 2.11, the null hypothesis was that internalization does not differ between liposomal samples. The alternative hypothesis was that they differ from each other.

## 3. Results

### 3.1. Characteristics of the Liposomes

**Figure 1:**
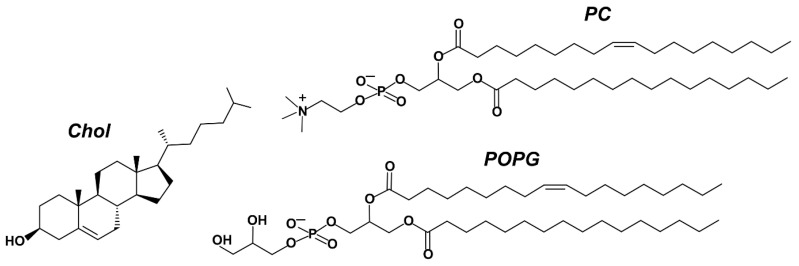
Structures of lipids used for liposome formulation: Chol, cholesterol; the most represented molecular species in egg phosphatidylcholine (PC) [38], palmitoyloleoylphosphoatidylcholine; and palmitoyloleoylphosphatidylglycerol (POPG).

Liposomes for this study (see Table 1 for compositions and sample designations) were made from cholesterol (Chol) and two phospholipids: phosphatidylcholine (PC) and phosphatidylglycerol (POPG) (Figure 1). Both egg yolk PC and POPG were characterized by low phase transition temperature (*T*_m_ egg PC is (–10) – (–5) °C and *T*_m_ POPG, –2 °C [36]). At room temperature, they formed fluid-phase lipid bilayers. When cholesterol was added in sufficient amount (>30% by mole), liquid ordered phase was formed, which is thicker and characterized by restricted mobility of hydrocarbon chains [37]. PC is a zwitterionic molecule, so the electrokinetic potentials of pure PC or PC–Chol (sample 33CH) liposomes were slightly below 0 (Table 1). The addition of POPG, whose phosphate group had p*K*_a_ of ~3, led to a substantial increase in the negative charge of liposomes in comparison with PC and 33CH. The five samples studied in the work covered different combinations of liquid ordered and disordered phases of neutral and negatively charged lipids, with liquid disordered phase studied at two anionic lipid ratios, 10 and 40%.

**Table 1:** Liposome compositions, average diameters, and zeta potential values.

Composition	Sample ID	DH ± SD, nm	PDI ± SD	Zeta Potential * ± SD, mV	
				Liposomes	Liposome–Protein Complexes
ePC	**PC**	117.4 ± 3.1	0.050 ± 0.016	–2.7 ± 0.4	–3.4 ± 0.2
ePC–Chol 67:33	**33CH**	129.7 ± 1.8	0.082 ± 0.010	–4.1 ± 1.1	–6.6 ± 0.6
ePC–Chol–POPG 57:33:10	**CHPG**	121.7 ± 0.5	0.058 ± 0.001	–49.1 ± 3.0	–33.4 ± 1.7
ePC–POPG 90:10	**10PG**	114.9 ± 1.1	0.059 ± 0.005	–41.0 ± 1.0	–33.5 ± 0.9
ePC–POPG 60:40	**40PG**	109.9 ± 2.4	0.058 ± 0.041	–64.1 ± 4.2	–22.3 ± 0.9

* See Section 2.3 and Section 2.4 for details regarding sample preparation for DLS and zeta potential measurements.

When liposomes were incubated with 50% human plasma and the resulting liposome–protein complexes were separated from free proteins, changes in liposome zeta potentials were observed (Table 1). For almost neutral PC and 33CH, the electrokinetic potential shifted moderately towards higher negative charge. In contrast, for formulations containing POPG, the absolute values of electrokinetic potential considerably decreased. Most plasma proteins are known to be negatively charged at physiological pH, and the protein corona on nanoparticles—regardless of the initial surface charge—typically confers zeta potential of around −20 mV thereon [39,40]. Negatively charged liposomes are known to adsorb more proteins when in contact with plasma compared with neutral ones [41]. Thus, the extent of zeta potential normalization in the presence of plasma proteins could be indicative of the amount of proteins bound, particularly those proteins relying on electrostatic interactions to bind the lipid bilayer.

### 3.2. Liposome Stability Assessment

Protein deposition on the surface of liposomes has several consequences regarding liposome stability [41]. First, proteins on the surface of liposomes can induce the aggregation of liposomes accompanied by the increase in the hydrodynamic diameter and sedimentation of the dispersions. Second, the osmotic pressure created by the concentrated protein solution results in the bilayer shrinkage and water escape from liposomes through the transient pores in the lipid bilayer. Depending on the bending rigidity of the bilayer, stability, and the amount of pores, this process, as such, can result in the complete rupture of the vesicles [30]. Lastly, some proteins might protrude or embed into the liposome membrane, thus destabilizing the bilayer and creating pores themselves, which leads to a loss of inner volume load (either a drug or a dye) [42,43].

**Figure 2:**
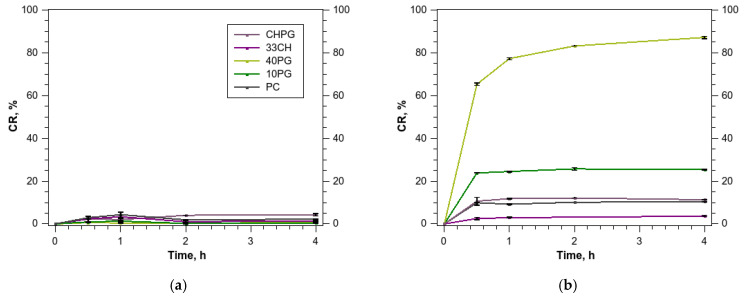
Calcein release assessment in PBS (**a**) and in 50% human plasma (**b**).

According to dynamic light scattering (DLS), we did not observe aggregation in studied samples (see Table S1). We estimated our samples’ stability in PBS and in 50% human plasma under physiological temperature (37 °C) using the calcein dye release assay. All liposomes with entrapped calcein were stable in the buffer (Figure 2a). Incubation of the liposomes with 50% plasma caused pronounced calcein leakage during the first hour of incubation, which then plateaued. The extent of leakage depended on the liposome composition.

In the liquid disordered bilayers, increasing quantities of POPG made the liposomes more susceptible to protein destabilization (Figure S1). When PC liposomes were supplemented with 10% of POPG, it doubled the initial (prior to plateau) loss of calcein (Figure 2b); 40% POPG in the bilayer increased the initial loss six-fold. The palmitoyloleoyl phosphatidylcholine (POPC)–POPG (60:40 by mole) bilayers should withstand higher osmotic pressures than pure POPC ones [44]. The loss in bilayer stability, therefore, could be attributed to specific changes in protein corona composition provoked by the phosphatidylglycerol polar region.

According to Janosi and Gorfe [45], for a POPC bilayer with ~23% of POPG, the anionic lipid enhances the polarization and order of surrounding solvent but does not affect the overall structure of the bilayer and chain order. When mixed with phosphatidylethanolamine, POPG decreases the membrane permeability, as observed in bacterial membranes [46]. These observations can be attributed to formation of intra- and intermolecular hydrogen bonds reported in computational studies for POPG and its mixtures with POPC and PE, which, in turn, should be accompanied by a decrease in area per lipid for POPG compared to POPC [45,46,47]. In such a case, the attraction of cationic proteins that favor patches of negatively charged lipids in the membrane through electrostatic interactions might cause considerable deformation of the bilayer. There are experimental data, however, that contradict computational findings and suggest that the area per lipid increases in POPG bilayers, presumably due to the repulsion rising between the charged headgroups [48]. In either case, the effect of charge-driven interaction with plasma proteins seemed to overdrive the effects of intrabilayer bond modulation upon changes in the bilayer composition, since the effects of liposome composition on dye release in PBS were negligible (Figure 2a).

The addition of cholesterol to the phospholipid bilayers helped to reduce the protein impact on their stability approximately two-fold. Again, this increase in the stability should probably be both due to the membrane transition to liquid ordered phase and due to the changes in the protein corona components associated with the cholesterol-containing liposomes.

### 3.3. Plasma Protein Binding by the Liposomes

To this day, there have been no reports on a method to separate dispersed (not surface-bound) liposome–protein complexes from the other plasma components (lipoproteins, immune complexes, protein aggregates) without contamination and with high yield [41,49]. To estimate the quality of the protein corona separation by size-exclusion chromatography (SEC), we used the control plasma sample treated in the same manner as the liposome–protein mixtures. Upon the SEC separation of the plasma control, protein concentration in fractions corresponding to the liposome elution was too low for a proteomics analysis. We combined the material of three SEC elutions for each control sample. The total protein amount in delipidated samples was too low to allow for a protein assay; we assayed peptide concentrations in hydrolysates instead (Figure S2). Even the triple plasma sample contained less peptides than fractions obtained upon liposome isolation, according to the Pierce assay (Figure S2). Nevertheless, major proteins contained therein could co-elute with the liposomes without any specific or non-specific interaction with the particles. Here, we termed proteins that were eluted together with the liposomes either upon binding to liposomes or merely in parallel as *protein corona*.

**Figure 3:**
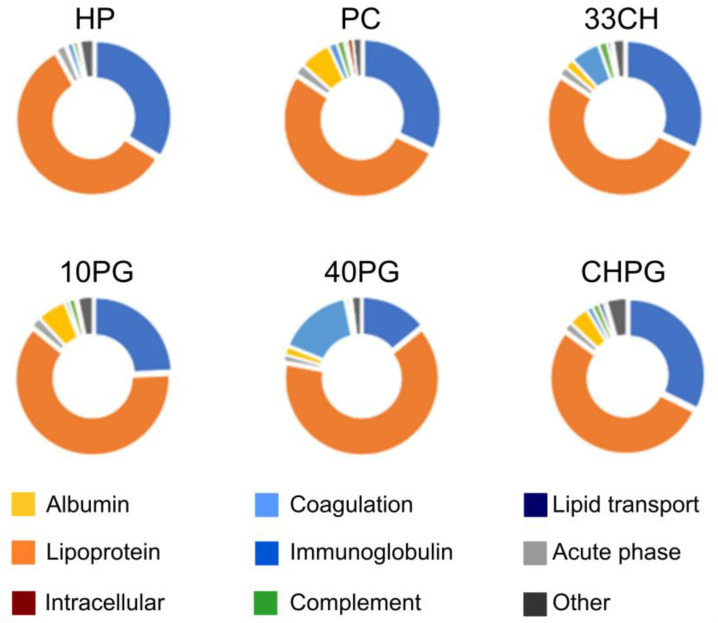
Relative abundance of functional groups of proteins with RPA > 0.1% in the protein coronas of the liposome samples and plasma control.

An increasing fraction of the negatively charged lipid (POPG) in fluid-phase liposomes correlated with a redistribution of protein corona proteins in favor of the lipoproteins and coagulation proteins instead of immunoglobulins (Figure 3 and Table 2). The addition of cholesterol led to lower albumin and higher coagulation protein recovery in the liposome coronas. Proteins associated with the CHPG sample, which had both 10% POPG and 33% cholesterol by mole, reflected the combined action of these effects.

According to the LFQ intensities (see Table S2), when compared with the plasma control, albumin and paraoxonase 1 were enriched in all liposome samples; IgG Fc-binding protein was depleted from all coronas compared to plasma. Apolipoprotein M was enriched on all samples except for 40PG. Immunoglobulin G heavy chain γ1, serotransferrin, hemopexin, and plasma protease C1 inhibitor were enriched on non-cholesterol samples; CD5 antigen-like protein and haptoglobin were depleted thereof. Apolipoprotein E, J chain, apolipoprotein A4, and ficolin-3 were depleted from liposomes with POPG and without cholesterol. Below, we discuss the highly abundant and differentially adsorbing proteins in greater detail.

#### 3.3.1. Immunoglobulins

Immunoglobulins are among the major components of human plasma. IgG and IgM (13.5 and 1.5 mg/mL, respectively) can initiate activation of the classical pathway of complement and be recognized by the opsonic receptors on phagocytes when adsorbed onto liposomes [50].

Immunoglobulin G (~150 kDa), represented primarily by the physiologically most abundant heavy chain variants IGHG1 and IGHG2 (Table 2), was the most abundant in the PC and 10PG liposomes among all samples. Increasing quantities of POPG correlated with lower IgG RPA values. As for the addition of cholesterol, CHPG had less IgG in the corona when compared to 10PG or PC, whereas it was absent from the top 25 proteins of the 33CH sample.

Immunoglobulin M (IgM; MW~900 kDa) was the second and fourth abundant protein in the liposome coronas; it was also detected in all liposome–protein corona samples by WB (Figure S3a). IgM fragments were present in abundance in the list of proteins eluted in negative control plasma fractions corresponding to liposome elution, because of the large size of the IgM pentamers. It might well be a contaminant in the liposome samples. According to the LFQ intensities, components of the IgM complexes were significantly depleted from the 40PG (IGHM and J chain) and 10PG (J chain) samples compared to control plasma, indicating the possibility of separation of the immune complexes from the liposomes under the experimental conditions. On the other hand, for cholesterol-containing samples, we did not obtain the same result from the LFQ intensities. Moreover, according to WB (Figure S3a), IgM may have concentrated on the 33CH and CHPG samples. Thus, IgM pentamers could not only co-elute with the cholesterol-containing liposomes but also bind the bilayer as well. IgM is frequently observed in the coronas of liposomes, including the plasma samples obtained from patients treated with DOXIL [25]. IgM adsorption is an issue for PEGylated drugs (including DOXIL), because anti-PEG antibodies—the prevalence of which rose from 0.2% in 1984 to 97.5% in 2019 in treatment-naïve individuals [51]—cause rapid elimination of the drug from bloodstream, as well as may result in adverse effects associated with complement activation [17,18].

Interestingly, immunoglobulin M is not only the first line of defense in reaction to unknown antigens but also a transporter molecule for another protein, the CD5-like protein (CD5L, otherwise called apoptosis inhibitor of macrophages). Each pentamer binds and stabilizes a single AIM/CD5L molecule [52]. CD5L is expressed mostly by the tissue macrophages and epithelial cells in the lung and helps to protect macrophages from apoptosis induced by different pathogens [53]. This association with IgM, along with its rather high abundance in the negative control, implies that CD5L could also co-elute with liposomes rather than selectively bind to them.

#### 3.3.2. Lipoprotein Components

The SEC method we used, with sufficient excess of the gel phase over the separated mixture (1:100 by volume), should certainly allow for separation of smaller lipoprotein particles, such as HDL, while there is a high chance that a fraction of larger ones remains in the same fractions as the liposome–protein complexes. Thus, apolipoproteins, if present among the liposome-associated proteins in appreciable amounts, could either be contaminants (especially when no difference with plasma is observed or the content is lower than that in plasma) or interact specifically with the liposomes either by themselves (for exchangeable apolipoproteins) or within the whole lipoprotein particle [54].

Apolipoprotein B was detected in all samples, but due to the fact that it is not an exchangeable apolipoprotein and is a major component of large VLDL particles (~30% ApoB100 by mass), chylomicrons (CM, contain ApoB48), and CM remnants, which overlap in size and density with the liposomes and, thus, co-elute with them during SEC, ApoB is highly likely a contamination [49]. Smaller LDL particles (~90% ApoB100 to total protein by mass) hold an intermediate position, that is SEC cannot guarantee separation thereof.

The protein components of HDL included, first of all, apolipoprotein A1 (80% of HDL protein by mass), as well as paraoxonase 1 (PON1), alpha-1-antitrypsin (SERPINA1), and serum amyloid A proteins (SAA4) [55]. ApoA1 is a freely exchangeable protein. It is also a minor component of chylomicrons and less than 10% of all ApoA1 exists in plasma in free or low-lipid bound form [56]. Only the 40PG sample was significantly depleted from ApoA1, suggesting that its presence among the liposome-associated proteins was probably a result of contamination. Western blotting yielded similar results, as we observed comparable low intensity bands for the samples, with slight signal increase in the case of CHPG (Figure S3b)

Paraoxonase (PON1) is a 43 kDa glycoprotein, an inhibitor of lipid oxidation and cholesterol biosynthesis in macrophages [57]. PON1 preferred liposomes without cholesterol. Its levels were significantly elevated in the protein coronas of PC and 10PG, where PON1 made it into the top ten, and also of 40PG, where PON1 made it into the top 15 proteins (Table 2). As for the CHPG sample, paraoxonase was evidently a part of the protein corona but not as abundant as in previously discussed samples. The presence of PON1 in the coronas of graphene nanorods has been shown to correlate positively with the secretion of IL-1β and IL-6 by macrophages upon the uptake of the nanoparticle−protein complexes [58].

ApoF is a heavily glycosylated (~40%), freely exchangeable, ~30-kDa protein found on a specific 470 kDa subset of HDL particles and LDL [59]. Along with ApoC1, it inhibits the function of cholesteryl ester transfer protein (CETP) on LDL. We observed ApoF deposition on all the liposomes, except for 40PG, compared to plasma control. Both cholesterol addition and an increase in the PG content reduced the significance of ApoF binding to liposomes.

Cathelicidin (CAMP or hCAP-18) is a 18-kDa proprotein, which yields an active antimicrobial peptide, LL-37. At physiological pH, LL-37 is a cationic α-helical peptide that disrupts bacterial membranes. Cathelicidin’s plasma level is 1.2 μg/mL. Sorensen and colleagues showed that about 20% of plasma CAMP is bound to the ApoA1-containing HDL particles and the rest are bound to the ApoB100-containing LDL and VLDL [60]. Thus, lipoproteins could be the source of cathelicidin for our liposomes. The protein coronas of POPG-containing liposomes were significantly enriched with CAMP in comparison with treated plasma. Yang and co-authors previously detected CAMP in the protein corona of DOPC–DOPG–Chol (1: 1: 1) liposomes, which differed from the CHPG sample by the lower PG content and lower degree of acyl chain saturation in the latter case [61]. According to the CAMP biological function, or rather that of its active form, LL-37, it can affect the stability of the PC and PC–PG bilayers [62,63]. This could contribute to the higher calcein release from the PG-containing liposomes.

Mature ApoC1 was the smallest (6.6 kDa) and the most positively charged (pKa 7.93) apolipoprotein. Its plasma concentration was ~60 µg/mL. It is an exchangeable apolipoprotein and, thus, a constituent of chylomicrons, VLDL, and HDL. ApoC1 was the most abundant protein in 40PG protein corona probably through electrostatic interactions and contributed to the high apolipoprotein content therein (Figure 3). Its appearance in the 33CH top ten proteins could be somewhat related to the function of the cholesteryl ester transfer protein (CETP) (ApoC1 is known to be a CETP inhibitor [64]), with further enrichment in the CHPG corona due to the contribution of the electrostatic effect (Table 2).

The corona of the 40PG liposomes was also found to be enriched with ApoC3 and ApoA4, which were demonstrated to reduce the uptake of polystyrene nanoparticles by human mesenchymal stem cells (hMSC) [65]. ApoC3 is a 9-kDa exchangeable apolipoprotein inhibiting the hepatic uptake of VLDL and chylomicron remnants [66]. The majority of the 44-kDa ApoA4 molecules, on the other hand circulated in plasma in free form, while some were part of chylomicrons and HDL. Thus, these proteins could be adsorbed onto 40PG independently of the liposome–lipoprotein interactions.

#### 3.3.3. The Coagulation Cascade

The second most abundant protein in the 40PG corona was beta-2-glycoprotein 1 (beta-2-GP1), formerly known as apolipoprotein H (ApoH), which is involved in multiple coagulation-related cascades. Its deposition was corroborated by WB (Figure S3c). Interestingly, beta-2-GP1 is a soluble protein, while it was shown to form aggregates when binding anionic membranes via a stretch of positively charged amino acids (protein sequence positions 282–287), Lys-Asn-Lys-Glu-Lys-Lys [67]. Yang and co-authors previously detected ApoH deposition on liposomes with high PG content (33 and 50%) [61]. In the work of Ritz and co-authors [65] conducted on polystyrene nanoparticles, the protein corona enrichment with beta-2-glycoprotein 1 led to higher cellular uptake of the nanoparticles by the hMSC.

Another coagulation protein enriched in the 40PG protein corona was the coagulation factor V (FV). It is a 330-kDa, heavily glycosylated protein with plasma concentration of ~10 μg/mL. Together with factor Xa, Ca^2+^, and anionic phospholipid membrane, FVa formed the prothrombinase complex. In an early work of Cutsforth and co-authors [68], FVa was shown to associate with PG-containing vesicles; the affinity of the interaction increased together with the PG content. Dissociation constant for the POPC vesicles with 35% PG was ~40 times lower than for 10% PG in POPC. Our data agree with these findings. Due to the dramatic difference in dissociation constants, we did not observe factor V on liposomes with lower PG content (10PG and CHPG) or without PG. For the 40PG sample, the ability of FV to penetrate anionic membranes could contribute to the calcein release that we observed in plasma (Figure 2b).

A thrombin substrate and the most abundant coagulation protein in plasma, fibrinogen, was in the top 25 proteins only for the 33CH liposomes (Table 2). Fibrinogen is a 340-kDa glycoprotein with plasma concentration range 1.5–3.5 mg/mL [69]. Faizullin and co-authors [70] previously observed selectivity in fibrinogen binding to PC liposomes, which manifested itself in preferable protein binding to solid DPPC liposomes over fluid PC.

#### 3.3.4. Complement Components

Factor C3 (~190 kDa) was the most abundant protein of the complement system with plasma levels of 1–1.5 mg/mL. When it was cleaved by the C3H_2_OBb convertase, the C3b part (~176 kDa) covalently bound nucleophilic groups on the surface in the vicinity, and the small C3a anaphylatoxin molecule (~9 kDa) was released. C3b, as well as the products of its further degradation, is recognized by the neutrophils, monocytes, dendritic cells, B cells, and NK cells [71]. Thus, C3 in the protein corona may promote phagocytosis of drug delivery system once it is recognized by these cells. Meanwhile, it was enriched only in the protein coronas of the PC sample.

Plasma protease C1 inhibitor (C1-INH) is another protein of the serpin superfamily SERPING1 (105 kDa, ~0.25 mg/mL in plasma). It inhibits factor XIIa and kallikrein of the contact system; coagulation factor XI, plasmin, and tissue plasminogen activator (t-PA) of fibrinolytic system; and, lastly, C1r, C1s, MASP-1, and MASP-2 of the complement, thus preventing the activation of the classical and lectin pathways [72,73]. Moreover, C1-INH can reversibly bind C3b and interfere with the C3b–factor B interaction, which is essential for the alternative pathway activation. Protein coronas of all our samples were enriched with C1-INH, except for the 33CH sample.

In order to suppress the involuntary activation of the complement cascade, inhibitory proteins factor H for the alternative pathway and C4b-binding protein (C4BP) for the classical pathway are expressed. The latter is a major effector protein with plasma levels of ~0.25 mg/mL. This protein consists of seven identical α-subunits (75 kDa) and one β-subunit (45 kDa), which gives the overall molar mass of about 500 kDa [74]. However, as it was in the top 25 proteins in the control sample, we could expect at least partial coelution of that protein. The only liposome sample with C4BP RPA values higher than that of the control was CHPG.

#### 3.3.5. Transport Proteins

Albumin (HSA, 66.5 kDa, ~40 mg/mL) is a major plasma protein; it is involved in the transport of fatty acids and other ligands. HSA is a highly flexible protein, which can change its structure in order to find the most advantageous conformation for an interaction [41]. Protein coronas of all liposome compositions were enriched with albumin in comparison with the plasma control. In Table 2 and Figure S3d, we can see that PC, 10PG, and CHPG boast higher content of albumin than the 33CH and 40PG samples. According to the Vroman effect theory, albumin can be forced out from the liposome corona by proteins with higher affinity to the membrane. Although albumin in the protein coronas is frequently regarded as a dysopsonin, the evidence supporting this is contradictory [75]. Particularly, gp18 and gp30 were identified as receptors that can mediate endocytosis of LNPs of certain compositions through surface-bound albumin [76].

Transferrin is a 79-kDa protein; it circulates in plasma at concentration of 2.5–3.0 mg/mL [77]. Transferrin is responsible for the extracellular transport of iron to actively dividing cells, including cancer cells. Transferrin-conjugated liposomes showed increased tumor uptake [78]. Here, transferrin was enriched in the protein coronas of the same set of liposomes as was albumin, i.e., PC, 10PG, and CHPG.

Hemopexin (HPX), a 60-kDa glycoprotein with plasma concentration range of 0.4–1.5 mg/mL, is another protein involved in iron homeostasis. Hemopexin binds free heme in circulation and transports it to the liver. It was found in coronas of the PC, 10PG, and CHPG samples. HPX binding to liposomes was detected for another formulation, doxorubicin-loaded liposomes Caelyx. In the work of Hadjidemetriou et al., HPX was among the proteins in coronas obtained ex vivo from six ovarian carcinoma patients with average RPA values ~ 0.2% [25].

Thus, we can conclude that both the negative charge and lipid phase influence the corona compositions. Electrostatic interactions should underlie the interactions between the PG-containing liposomes and such cationic proteins as ApoC1 and beta-2-glycoprotein 1. Fluidity of the bilayer is probably related to its capacity to adapt the interface of interaction with bound proteins to the protein structure through lateral mobility of lipids and flexibility of individual molecules. The better the bilayer adapts, the more protein it can bind. Overall, the mass spectrometry data on the composition of liposome protein coronas reflects the literature reports of studies conducted under a variety of conditions and utilizing diverse experimental techniques evidencing robustness of the conclusions we made on the differences between the samples. The overview of functions and roles of the proteins differentially adsorbed on studied liposomes suggests that there may be differences in the cell uptake of the liposomes.

**Figure 4:**
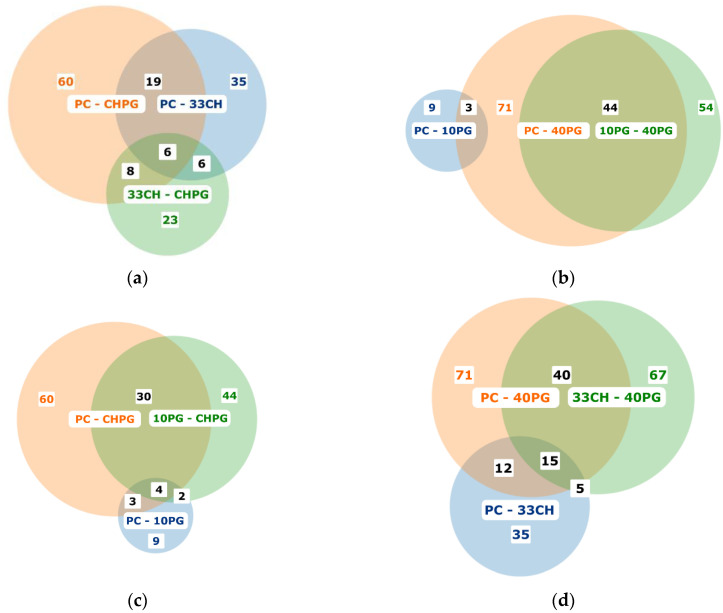
Venn diagrams depicting proteins differentially detected in pairs of liposome sets: (**a**) PC vs. 33CH, PC vs. CHPG, and 33CH vs. CHPG; (**b**) PC vs. 10PG, PC vs. 40 PG, and 10PG vs. 40PG; (**c**) PC vs. CHPG, PC vs. 10PG, and 10PG vs. CHPG; and (**d**) PC vs. CHPG; PC vs. 40PG, and 33Ch vs. 40PG. See Table S3 for the lists of differentially adsorbed proteins. Colored figures indicate the total number of significantly different proteins for the indicated pair; black figures indicate the number of significantly different proteins share by two or three pairs.

The Venn diagrams (Figure 4) illustrate the relative magnitude of differences in the protein coronas of different liposomes, which, in turn, can be interpreted together with the stability thereof in the presence of plasma proteins (Figure 2b). The least number of differentially adsorbed proteins was observed between the PC and 10PG samples (nine proteins; Figure 4b,c). These included ApoC1, cathelicidins, and beta-2GP1, all positively charged at physiological pH. They were the candidate proteins destabilizing the 10PG bilayer significantly compared to PC. The highest number of differentially adsorbed proteins was observed in the PC vs. 40PG pair (71; Figure 4b,d). The proteins specific for the 40PG bilayer resulted in the lowest stability of the liposomes. The number of differentially adsorbed proteins in the PC vs. CHPG group (60; Figure 4a,c) was also high, and exceeded that of the PC vs. 33CH pair (35; Figure 4a,d). These proteins, specific to cholesterol-containing bilayers, imparted the 33CH liposomes with even a greater stability than that of a plain PC bilayer and probably compensated for the effects of PG-associated proteins, resulting in the CHPG stability being close to that of PC.

### 3.4. Liposome Internalization by the Cells

#### 3.4.1. Cultured Cells

**Figure 5:**
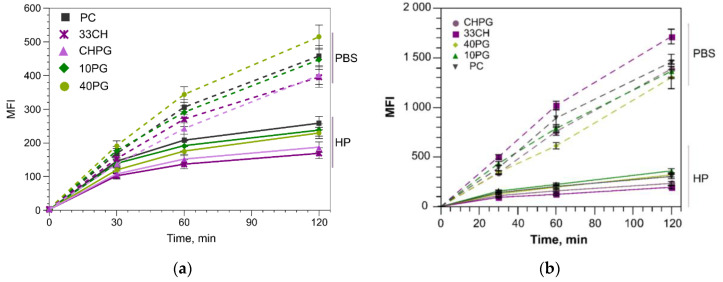
Liposome uptake by EA.hy926 (**a**) and U937 (**b**) cells. Liposomes (100 µM total lipids) in PBS or with protein corona formed in human plasma (HP) were incubated with cells and analyzed by flow cytometry. Representative experiment, mean fluorescence intensity (MFI) ± SD.

Protein coronas offer multiple ligands (e.g., HSA, ApoH, ApoE, transferrin) that could promote liposome interaction with the cells in various manners. However, as demonstrated by Figure 5, there were no significant effects of plasma proteins on the liposome consumption by endothelial (target) EA.hy926 and pro-monocytic (potential off-target) U937 cultured cells. One of the reasons could be the overall low liposome uptake by the cells in the presence of plasma. On the other hand, there is growing evidence that in the process of nanoparticle–cell interaction, the cell features contribute to the intensity of the uptake not less than the nanoparticle, in our case liposome, features do, with subtle differences in the liposome surface chemistry poorly sensed by the cells [79]. What matters more is the source of the proteins that form the protein corona, if any (Figure S4 compares PBS, FBS, HP), and whether the liposome–protein complexes are presented to the cells alone or in the presence of other proteins. In this aspect, our data agree with those of Yang and co-authors [80].

#### 3.4.2. Ex Vivo Cells

**Figure 6:**
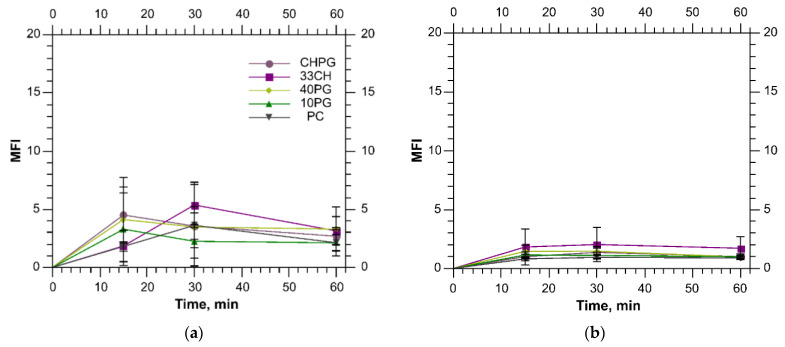
Liposome uptake by monocytes (**a**) and neutrophils (**b**) from human blood.

When studying liposome interactions with the blood cells, we were interested in the phagocytic cells: monocytes and neutrophils. We eliminated the erythrocytes by lysis. The lymphocytes did not internalize the liposomes reflecting their low phagocytic capacity [81]. The liposome internalization by both monocytes and neutrophils from human blood was negligeable (Figure 6).

The liposome endocytosis by the blood phagocytes was mediated by the receptors of plasma proteins associated with the liposomes. For example, complement component C3 and immunoglobulins IgG are considered to be the opsonins that attract phagocytes [41]. Complement components CR1, CR3, and CR4, and the FcγR I–III receptors for the constant regions of IgG, as well as C1q (for monocytes), and C1-INH and vitamin D binding protein (for neutrophils) could also contribute to the recognition of the liposomes by the blood phagocytes [82].

**Figure 7:**
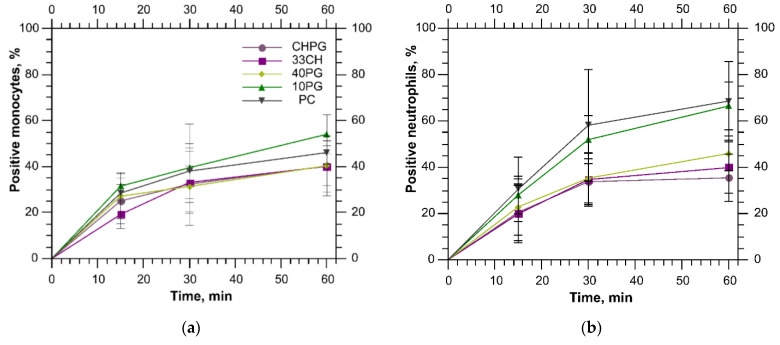
The fraction of positive cells that internalized the liposomes in the monocyte (**a**) and neutrophil (**b**) populations.

According to Table S2, both the complement component C3 and IgG were of relatively low abundance in the protein coronas of all our samples. Low opsonization could be the reason for the low liposome uptake by both monocytes and neutrophils. Interestingly, even though overall liposome internalization was low, roughly half of the monocyte population was engaged into phagocytosis (Figure 7a). Neutrophils exhibited similar or even more active behavior in terms of liposome recognition (Figure 7b). It appears that monocytes absorbed all liposome samples equally well. Monocyte uptake may have been evened by clusterin (Table S2) binding to all samples. As for neutrophils, it appeared that cholesterol addition into the bilayer decreased the number of neutrophils prone to phagocytosis of liposomes (Figure 7b). After 1 h of incubation in the blood, the percentage of positive neutrophils for 33CH and CHPG was lower than for PC and 10PG by one third (with *p* values < 0.04). The addition of cholesterol to 10PG produced the same effect as further increase in POPG content in the liposomes (40PG). This trend corresponds to the results of the mass-spectrometry analysis as C1-INH, which is recognized by neutrophils, has higher abundance in the protein coronas of the PC and 10PG samples. Proteins that were preferably bound by the 40PG sample, although they drastically affected its stability, did not have such a pronounced effect on the internalization by the cells.

## 4. Conclusions

In the study, we showed that both the total amount of proteins and the number of protein species associated with the liposomes in plasma depend little on the bilayer composition thereof (liquid ordered or disordered phase of neutral or anionic bilayer). However, changes in the lipid composition led to a considerable shift in relative abundances of the proteins in the coronas, which affects liposome integrity in the presence of plasma proteins. We confirmed the positive effect of cholesterol in liposomes on their stability in plasma. Cholesterol somewhat improved the liposome recognition by neutrophils as well, which at least partially depended on the protein corona composition. Overall, the effect of the differences in the lipid bilayer and protein corona compositions on the liposome uptake by cells was negligible.

Expanding the array of basic liposome compositions to investigate their protein coronas and correlating the results with the in vivo data should be the next steps of the research. Exploring the roles of individual proteins found to be differentially bound on the liposomes would also contribute to our understanding of the protein corona effects on liposome carriers and it would improve the design of the new ones.

## Figures and Tables

**Table 2:** Relative protein abundance values (%; mean ± SD) for the top 25 polypeptide chains in the protein coronas of the liposome samples and plasma control.

HP			PC			33CH			10PG			40PG			CHPG		
	Polypeptide Chain	RPA, % (Mean ± SD)		Polypeptide Chain	RPA, % (Mean ± SD)		Polypeptide Chain	RPA, % (Mean ± SD)		Polypeptide Chain	RPA, % (Mean ± SD)		Polypeptide Chain	RPA, % (Mean ± SD)		Polypeptide Chain	RPA, % (Mean ± SD)
**1**	APOC4-C2	29.2 ± 6.0	**1**	APOC4-C2	25.0 ± 5.6	**1**	APOC4-C2	25.2 ± 1.2	**1**	APOC4-C2	24.7 ± 6.2	**1**	APOC1	37.1 ± 5.6	**1**	APOC4-C2	22.5 ± 3.5
**2**	IGHM	12.4 ± 2.6	**2**	APOC3	10.8 ± 1.4	**2**	IGHM	12.3 ± 1.5	**2**	IGHM	8.8 ± 3.4	**2**	APOH	14.2 ± 4.9	**2**	IGHM	11.1 ± 2.1
**3**	APOE	9.5 ± 0.7	**3**	IGHM	10.1 ± 4.7	**3**	APOC3	9.4 ± 0.7	**3**	APOC3	8.2 ± 1.4	**3**	APOC4-C2	11.0 ± 1.2	**3**	APOC1	10.1 ± 1.3
**4**	APOC1	7.0 ± 2.3	**4**	APOE	7.8 ± 1.8	**4**	APOE	7.0 ± 1.5	**4**	APOC1	8.0 ± 3.1	**4**	IGHM	6.0 ± 1.5	**4**	APOC3	6.9 ± 1.7
**5**	APOC3	6.6 ± 1.7	**5**	ALB	6.3 ± 0.9	**5**	IGKC	5.8 ± 0.6	**5**	APOE	6.2 ± 0.8	**5**	APOE	5.6 ± 1.5	**5**	IGKC	6.7 ± 2.0
**6**	IGLC6	5.5 ± 1.8	**6**	APOM	4.3 ± 1.3	**6**	APOC1	5.4 ± 5.4	**6**	ALB	5.6 ± 0.9	**6**	APOC3	4.9 ± 0.7	**6**	APOE	6.6 ± 1.2
**7**	IGKC	4.6 ± 1.0	**7**	IGKC	3.8 ± 1.7	**7**	IGLC6	4.0 ± 1.4	**7**	IGKC	4.0 ± 1.0	**7**	IGLC6	2.2 ± 1.4	**7**	IGLC6	4.3 ± 0.7
**8**	IGJ	3.3 ± 1.1	**8**	IGLC6	3.4 ± 1.8	**8**	IGJ	2.3 ± 0.5	**8**	PON1	3.7 ± 1.5	**8**	ALB	1.7 ± 0.8	**8**	ALB	4.2 ± 0.9
**9**	CD5L	1.7 ± 0.7	**9**	PON1	2.2 ± 0.9	**9**	FGA	2.0 ± 0.0	**9**	IGLC6	3.2 ± 1.4	**9**	IGKC	1.4 ± 0.7	**9**	IGJ	2.6 ± 0.4
**10**	HP	1.4 ± 0.2	**10**	IGJ	2.1 ± 1.0	**10**	ALB	1.9 ± 0.8	**10**	APOM	2.0 ± 0.8	**10**	IGJ	1.4 ± 0.5	**10**	APOA1	1.9 ± 0.3
**11**	APOA2	1.3 ± 0.7	**11**	APOA1	1.5 ± 0.4	**11**	FGB	1.6 ± 0.0	**11**	IGJ	1.8 ± 0.7	**11**	CAMP	1.0 ± 0.4	**11**	CAMP	1.9 ± 0.7
**12**	APOA1	1.3 ± 0.3	**12**	APOF	1.4 ± 0.3	**12**	FGG	1.4 ± 0.0	**12**	CAMP	1.6 ± 0.9	**12**	APOA2	0.9 ± 0.4	**12**	CD5L	1.4 ± 0.2
**13**	VWF	1.1 ± 0.2	**13**	IGHG1	1.2 ± 0.3	**13**	CD5L	1.4 ± 0.3	**13**	APOA1	1.3 ± 0.3	**13**	PON1	0.9 ± 0.7	**13**	APOB	1.3 ± 0.1
**14**	IGHV3-74	1.1 ± 0.5	**14**	APOB	1.1 ± 0.2	**14**	APOA1	1.1 ± 0.2	**14**	C4BPA	1.2 ± 0.3	**14**	APOM	0.9 ± 0.4	**14**	APOA2	1.1 ± 0.1
**15**	APOB	0.9 ± 0.1	**15**	CD5L	1.0 ± 0.4	**15**	HP	1.0 ± 0.2	**15**	IGHG1	1.2 ± 0.5	**15**	CD5L	0.8 ± 0.3	**15**	HP	1.0 ± 0.2
**16**	IGKV2-24	0.7 ± 0.3	**16**	VWF	0.9 ± 0.2	**16**	APOB	1.0 ± 0.0	**16**	CD5L	1.0 ± 0.4	**16**	SAA2-SAA4	0.7 ± 0.2	**16**	APOD	0.9 ± 0.2
**17**	APOD	0.6 ± 0.3	**17**	HP	0.9 ± 0.2	**17**	VWF	0.8 ± 0.1	**17**	APOA2	1.0 ± 0.4	**17**	APOD	0.5 ± 0.2	**17**	VWF	0.8 ± 0.1
**18**	LGALS3BP	0.6 ± 0.1	**18**	APOA2	0.8 ± 0.4	**18**	APOM	0.7 ± 0.4	**18**	APOB	1.0 ± 0.3	**18**	APOA1	0.5 ± 0.2	**18**	IGHV3-74	0.8 ± 0.1
**19**	IGHV3-72	0.6 ± 0.3	**19**	IGHV3-74	0.7 ± 0.3	**19**	APOA2	0.7 ± 0.3	**19**	HP	0.9 ± 0.2	**19**	HP	0.5 ± 0.2	**19**	C4BPA	0.7 ± 0.2
**20**	SAA2-SAA4	0.5 ± 0.2	**20**	IGHV3-72	0.7 ± 0.5	**20**	IGHV3-74	0.7 ± 0.2	**20**	APOF	0.8 ± 0.2	**20**	VWF	0.5 ± 0.1	**20**	SAA2-SAA4	0.7 ± 0.1
**21**	IGKV4-1	0.5 ± 0.2	**21**	SAA2-SAA4	0.6 ± 0.2	**21**	CLU	0.7 ± 0.1	**21**	VWF	0.7 ± 0.1	**21**	IGHG1	0.4 ± 0.1	**21**	IGHA1	0.6 ± 0.3
**22**	IGLV8-61	0.4 ± 0.2	**22**	C4BPA	0.6 ± 0.2	**22**	SAA2-SAA4	0.7 ± 0.2	**22**	IGHA1	0.7 ± 0.2	**22**	IGK	0.4 ± 0.3	**22**	APOM	0.6 ± 0.1
**23**	C4BPB	0.4 ± 0.3	**23**	SERPINA1	0.6 ± 0.1	**23**	C4BPA	0.6 ± 0.2	**23**	SAA2-SAA4	0.7 ± 0.2	**23**	APOB	0.3 ± 0.0	**23**	IGK	0.6 ± 0.1
**24**	ALB	0.4 ± 0.1	**24**	IGK	0.6 ± 0.2	**24**	IGK	0.6 ± 0.3	**24**	IGHG-2	0.6 ± 0.2	**24**	IGLL5	0.3 ± 0.1	**24**	IGHG1	0.5 ± 0.2
**25**	IGHA1	0.4 ± 0.3	**25**	IGHG2	0.5 ± 0.1	**25**	APOD	0.5 ± 0.2	**25**	IGHV3-74	0.6 ± 0.3	**25**	F5	0.3 ± 0.0	**25**	IGKV2-24	0.5 ± 0.2

## Data Availability

The data presented in this study are available in Supplementary Materials.

## Supplementary Materials

The following supporting information can be downloaded at: https://www.mdpi.com/article/10.3390/membranes13070681/s1. Table S1: Liposome diameter before incubation with 50% HP and after incubation followed by separation of the liposome−protein complexes using SEC; Table S2: LC-MS/MS iBAQ and LFQ intensities; Table S3: Statistically significant differences in associated proteins; Figure S1: Calcein release assessment in PBS (a) and in 50% human plasma (b) for liposomes with increasing POPG percentage; Figure S2: Peptide content in samples after proteolysis and filtration on Strap filters; Figure S3: Western blotting results with (a) anti-IgM, (b) anti-ApoA1, (c) anti-beta-2-glycoprotein 1, and (d) anti-has antibodies; Figure S4: Liposome (33CH and CHPG samples) uptake by EA.hy926 cells without protein corona, with protein corona formed in FBS and with protein corona formed in HP.
