# Supplementary material for: Protein Corona of Anionic Fluid-Phase Liposomes Compromises Their Integrity Rather than Uptake by Cells
Source: Membranes (Basel). 2023 Jul 20;13(7):681. doi: 10.3390/membranes13070681 (PMC10384875; doi:10.3390/membranes13070681)
Supplement: Supplementary file 1 [file membranes-13-00681-s001.zip › membranes-2458963-supplementary.pdf]

Supplementary Information

Protein Corona of Anionic Fluid-Phase Liposomes Compromises Their Integrity rather than Uptake by Cells

Daria Tretiakova [1], Maria Kobanenko [1], Anna Alekseeva [1], Ivan Boldyrev [1], Sergey Khaidukov [2], Viktor Zgoda [3], Olga Tikhonova [3], Elena Vodovozova [1] and Natalia Onishchenko [1, a, *]

[1] Laboratory of lipid chemistry, Department of chemical biology of glycans and lipids, Shemyakin–Ovchinnikov Institute of Bioorganic Chemistry, Russian Academy of Sciences, ul. Miklukho-Maklaya 16/10, 117997 Moscow, Russian Federation; mkobanenko@lipids.ibch.ru (M.K.); daria@lipids.ibch.ru (D.T.); anna@lipids.ibch.ru (A.A.); ivan@lipids.ibch.ru (I.B.); elvod.ibch@yandex.ru (E.V.)

[2] Laboratory of carbohydrates, Department of chemical biology of glycans and lipids, Shemyakin–Ovchinnikov Institute of Bioorganic Chemistry, Russian Academy of Sciences, ul. Miklukho-Maklaya 16/10, 117997 Moscow, Russian Federation

[3] Institute of Biomedical Chemistry, ul. Pogodinskaya 10, 119121, Moscow, Russian Federation; vic@ibmc.msk.ru (V.Z.); ovt.facility@gmail.com (O.T.)

[a] The author's current affiliation is the Center for Soft and Living Matter, Institute for Basic Science, UNIST-gil 50 bldg. 103, 44919 Ulsan, Republic of Korea.

[*] Correspondence: natalia.r.onishchenko@gmail.com; Tel.: +8210-5855-5281

Table S1. Liposome diameter before incubation with 50% HP and after incubation followed by separation of the liposome−protein complexes using SEC

**Sample ID**	**D_H ± SD, nm**	
	Liposomes	Liposome−protein complexes
**PC**	194.4 ± 3.8	186.2 ± 1.8
**33CH**	199.9 ± 4.4	190.1 ± 3.0
**CHPG**	164.6 ± 2.6	150.3 ± 1.4
**10PG**	153.1 ± 0.6	160.6 ± 5.4
**40PG**	148.7 ± 1.1	232.2 ± 9.5

Data obtained for ~200-nm liposomes prepared in 10 mM KCl with 1 mM K_2HPO_4 and 1 mM KH_2PO_4 measured using Litesizer 500 (Anton Paar GmbH, Austria).

Calcein release assay

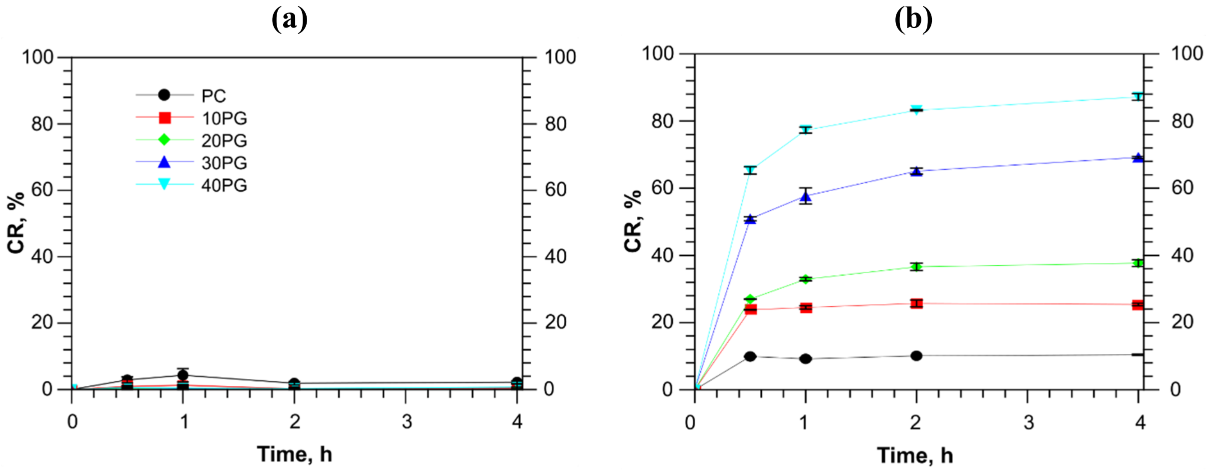

Figure S1. *Calcein release assessment in PBS (a) and in 50% human plasma (b) for liposomes with increasing POPG percentage.*

Total peptide assay

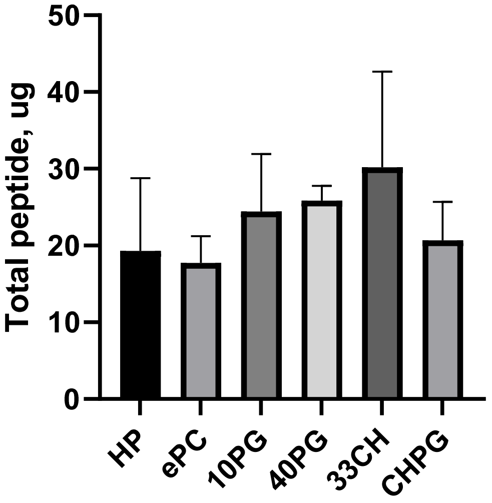

Figure S2. *Peptide content in samples after proteolysis and filtration on STrap filters measured using the Pierce™ Quantitative Colorimetric Peptide Assay kit (Thermo Scientific, Waltham, MA, USA). No statistically significant differences according to Welch's ANOVA, $p < 0.05$. Note that HP sample represents combined material of three SEC runs (one SEC run was sufficient for other samples).*

Western blotting

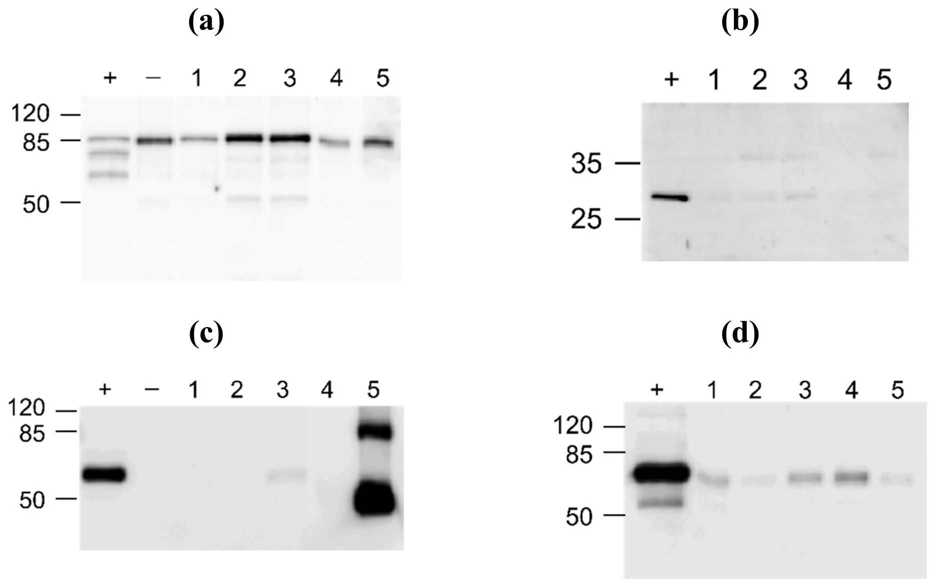

Figure S3. *Western blotting results with (a) anti-IgM, (b) anti-ApoA1, (c) anti-beta-2-glycoprotein 1, and (d) anti-HSA antibodies. Designations: +, diluted HP; -, negative control HP; 1, PC; 2, 33CH; 3, CHPG; 4, 10PG; 5, 40PG*

Western blotting has given us the reassurance of mass spectroscopy data, as we have observed the same patterns of protein adsorption for IgM, ApoA1, beta-2-glycoprotein 1, and albumin (Figs. S2a−d).

Liposome uptake by EA.hy926 cells

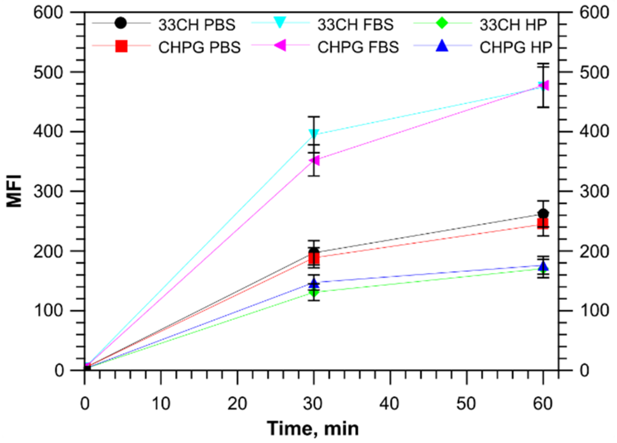

Figure S4. *Liposome (33CH and CHPG samples) uptake by EA.hy926 cells without protein corona, with protein corona formed in FBS and with protein corona formed in HP.*
